# The Effect of Type of Oil and Degree of Degradation on Glycidyl Esters Content During the Frying of French Fries

**DOI:** 10.1007/s11746-015-2715-3

**Published:** 2015-09-18

**Authors:** Magda Aniołowska, Agnieszka Kita

**Affiliations:** Department of Food Storage and Technology, Faculty of Food Science, Wroclaw University of Environmental and Life Sciences, Chelmonskiego 37 Str, 51-630 Wroclaw, Poland

**Keywords:** Glycidyl esters of fatty acid, LC–MS, Type of oil, Food analysis

## Abstract

The aim of the study was to determine the effect of oil degradation on the content of glycidyl esters (GEs) in oils used for the frying of French fries. As frying media, refined oils such as rapeseed, palm, palm olein and blend were used. French fries were fried for 40 h in oils heated to 180 °C in 30-min cycles. After every 8 h of frying, fresh oil and samples were analyzed for acid and anisidine values, color, refractive index, fatty acid composition, and content and composition of the polar fraction. GEs were determined by LC–MS. Hydrolysis and polymerization occurred most intensively in palm olein, while oxidation was reported for rapeseed oil. The degradation of oil caused increased changes in the RI of frying oils. Losses of mono- and polyunsaturated fatty acids were observed in all samples, with the largest share in blend. The highest content of GE found in fresh oil was in palm olein (25 mg kg^−1^) and the lowest content of GE was found in rapeseed oil (0.8 mg kg^−1^). The palm oil, palm olein and blend were dominated by GEs of palmitic and oleic acids, while rapeseed oil was dominated by GE of oleic acid. With increasing frying time, the content of GEs decreased with losses from 47 % in rapeseed oil to 78 % in palm oil after finishing frying.

## Introduction

Glycidyl esters of fatty acids (GEs) are process contaminants that are formed during the refining of vegetable oils and fats, as is the case with 3-monochloro-1,2-propandiol fatty acid esters (3-MCPD-esters). It seems that mono- and diacylglycerol may be the precursors of GE formed by an intermolecular mechanism under the conditions of high-temperature refining [1]. Toxicological investigations of glycidyl fatty acid esters are not yet available. However, the International Agency for Research on Cancer [2] classified glycidol as probably carcinogenic to humans. To date, it remains unclear to what extent glycidol is split off from ester-bound glycidol in the human body. For risk assessments, therefore, the worst-case scenario should be applied. In other words, it is assumed that GE is 100 % broken down into free glycidol in humans [3].

Deep frying is one of the most popular methods for the preparation of foodstuff, and has gained a high popularity both in restaurants and in industry because of its speed and operational simplicity. At temperatures between 140 and 180 °C, heat is transferred from the oil to the food, water is evaporated from the food and oil is absorbed in it, so a remarkable part of the oil is taken up by the product. Therefore, the sensory quality of the oil greatly influences the quality of the fried product. During deep frying, hydrolysis, oxidation, and polymerization of the oil takes place. Hydrolysis increases the amount of free fatty acids, mono- and diacylglycerols, and glycerol in oils. Oxidation occurs at an even greater rate than hydrolysis [4].

The ratio of oil degradation mainly depends on the type and quality of the oil, especially fatty acid composition. The selection of oils should be based on the optimization of the process with regards to culinary aspects as well as nutritional, physiological and technological requirements, but cost should not be the main decisive factor [5].

Palm oil is the most abundantly produced oil in the world. In 2014/2015, world supply and distribution of palm oil amounted to around 62 million metric tons [6]. Its use in the commercial food industry is widespread because of the lower cost of the refined product when used for frying. Solid fats are more stable at higher temperatures and have better oxidation stability than liquid oils with a high content of unsaturated fatty acids. After palm oil fractionation, palm olein is obtained. Palm olein is less saturated than palm oil and its use has increased because it has better frying properties as well as high stability and pleasant room odor during the frying process [7]. Palm olein is also commonly used by some food industries to produce French fries, which are a very popular fried product [8]. Although much less perceived than in palm oil, palm olein does impact a waxy or greasy mouth feeling to products prepared in it, especially in cold weather, affecting its overall acceptance. Furthermore, the high content of saturated fatty acids may pose an additional disadvantage for palm olein from nutritional point of view [9]. Due to the consistency of palm olein, requiring liquefaction before filling the fryer, frying fats are often prepared as mixtures of palm olein and high oleic refined oils. These products are called “new generation fats”. These types of frying fats have superior functional properties and preferred fatty acid composition [10]. Rapeseed oil, with a high level of unsaturated fatty acids which promote heart benefits, has a good nutritional profile. Based on its nutritionally well-balanced composition, rapeseed oil has become one of the most common vegetable oils in Poland and throughout middle Europe.

During deep frying, a considerable portion of the frying medium may be absorbed by the food. GEs may be present in any foods that contain refined vegetable oils and fats. There have been reports of GEs in a variety of refined oils, but there is a lack of research on changes in the content of these compounds during frying. So far, the highest levels of GEs have been found in refined palm oil [11]. It can be assumed that foods with a large share of palm oil also contain the highest levels of GEs.

Therefore, the objective of this research was to determine the effect of oil type and degree of degradation on the content of glycidyl esters.

## Materials and Methods

### Materials

The materials used were refined oils such as rapeseed (RO), palm (PO), palm olein (POn) and the blend (MIX), a mixture of palm olein with high oleic sunflower oil and rapeseed oil, together with frozen pre-fried French fries. The frying media were obtained from a local producer and packed in containers with a capacity of 10 L.

Pre-frozen French fries (made from one potato variety of identical technological parameters) were collected from the technological line of McCain Poland Manufacturers, immediately after stage I of frying. Stage II of frying was performed in the laboratory. Portions with a mass of 5 kg of product were packed in plastic bags. The material taken for the study consisted of French fries frozen at −18 °C prior to frying.

### Chemicals

For the determination of acid, peroxide, and anisidine values, chemicals of analytical grade were purchased. For fatty acids and polar fraction content and composition, the chemicals were of high-performance liquid chromatography (HPLC) grade, both from Merck (Darmstadt, Germany). In addition, methanol, acetonitrile, isopropanol, chloroform, acetone, ethyl acetate, diethyl ether, formic acid and *n*-hexane of HPLC grade were purchased from Sigma-Aldrich (St. Louis, MO, USA). Ultra-pure water was prepared using a Milli-Q purification system (Millipore, Bedford, MA, USA) and was used in all procedures.

Glycidyl palmitate (C16:0-GE) 98 %, stearate (C18:0-GE) 98 %, oleate (C18:1-GE) 98 %, linoleate (C18:2-GE) 90 % and linolenate (C18:3-GE) 85 % were purchased for liquid calibration solutions from Wako Chemicals (Neuss, Germany). Reverse-phase solid phase extraction (SPE) cartridges, Sep-Pak Vac C18 cartridges (500 mg) and normal-phase Sep-Pak Vac Silica cartridges (500 mg) were purchased from Waters (Milford, MA, USA). Internal standards [glycidyl palmitate (C16:0-GE-d5) 96 %, stearate (C18:0-GE-d5) 98 %, oleate (C18:1-GE-d5) 98 %, linoleate (C18:2-GE-d5) 98 % and linolenate (C18:3-GE-d5) 90 %] were purchased from Toronto Research Chemicals (Toronto, ON, Canada).

### Frying Procedure and Oil Sampling

The frying was simultaneously conducted in duplicate in 5-L capacity restaurant style stainless steel deep fryers (Beckers, Italy). A batch of 100 g of frozen French fries was fried for 4 min in refined oils heated at 180 ± 5 °C, which was monitored and kept constant. Frying was conducted in 30-min cycles for 8 h daily for five consecutive days. Total frying time was 40 h. At the end of each day of frying, the fryers were shut off, cooling to 60 ± 5 °C and all frying media were filtered to remove solid debris. Then, 100-mL samples of oil after filtration step were taken daily from each fryer (after 8, 16, 24, 32 and 40 h) and were kept frozen at −20 °C until analysis. After that, the oils were left to cool overnight. Every morning, the oils were replenished before the frying process to compensate for losses caused by the collection of samples and absorption of fat by French fries, adding fresh oil of a volume of less than 5 % of the total oil volume.

### Acid, Peroxide, and Anisidine Values

Acid value (AV), peroxide value (PV) and anisidine (AnV) value contents were determined in the frying media according to AOAC standards [12].

### Fatty Acid Composition

The fatty acids were methylated for analysis by gas chromatography (GC) based on the AOCS official method [13]. The resulting fatty acid methyl esters (FAME) were analyzed on a PU 4410 gas chromatograph (Philips, UK), using a capillary column RTX-2330 (Restek, USA) which was 105 m in length with a diameter of 0.25 mm i.d. and a film thickness of 0.2 μm. The detector (FID) and injection temperatures were 260 °C. The column temperature ranged from 160 °C (30 min) to 180 °C (17 min) at 3 °C/min and to 220 °C (15 min) at 5 °C/min. Helium was the carrier gas. The Star Chromatography Workstation (v.6.6) with software from Varian was used as the data handling system. The fatty acid composition was expressed as the percentage of total fatty acids.

Oil degradation during frying decreased the levels of polyunsaturated fatty acid and increased the saturated fatty acid content. The losses of polyunsaturated fatty acids (defined as changes to the unsaturation ratios of C18:2 and C16:0) in relation to the initial summary content of these fatty acids in fresh oils, were calculated according to Peer and Swoboda [14].

### Refractive Index

The refractive index (RI) of the oil samples was determined using a refractometer (Rudolph Research Analytical, model J157, USA) at 40 °C. Five repetitions were performed for each test.

### Color Analysis

The color of the fresh and used oils was determined using the Chroma Meter CR-400 system (Konica Minolta, Japan). The differences in the color of the samples were determined on the basis of a measurement in the CIE system at *L, a* and *b* configurations. The value Δ*E* was used to evaluate the accuracy and acceptability of the true and measured color differences. The total color difference (Δ*E*) was calculated using the following equation:

$$\Delta E = \sqrt {\left( {L_{0} - L_{f} } \right)^{2} + \left( {a_{0} - a_{f} } \right)^{2} + \left( {b_{0} - b_{f} } \right)^{2} }$$

where ‘*f*’ represents a thermally treated oil and ‘0’ is the initial oil.

### Total Polar Components (TPC)

The gravimetric method was used to determine the total amount of polar components after the column chromatography separation of the non-polar fraction, according to AOAC method 982.27 [15]. Two fractions were collected: the non-polar fraction that was eluted with a mixture of petroleum ether and diethyl ether (90:10 v/v for fresh oils and 87:13 v/v for oils after frying) and the polar fraction, which was removed with diethyl ether. The polar fraction was analysed for composition.

### Polar Fraction Composition

The polar fractions obtained above were analysed using high-performance size exclusion chromatography (HPSEC) following the method of Dobarganes et al. [16]. Separation was performed on a liquid chromatographic system, Varian (Paolo Alto, CA, USA), consisting of a ternary pump (230 Pro-Star) and an autosampler (430 Pro-Star). Components were separated on Phenogel columns SEC/GPC (300 × 7.8 mm; 100 and 500 Å) that were connected in series, and were equipped with a pre-column (50 × 7.8 mm) (Phenomenex, USA). An RI detector was used (Knauer, Germany). Using this system, the following compounds were separated and quantified: triglyceride polymers and dimers (TGP and TGD), oxidized triacylglycerols (oxTAG), diacylglycerols (DAG) and free fatty acids (FFA).

### Glycidyl Ester of Fatty Acids Composition

The GEs analysis was done on samples of frying oils as well on the fat extracted from the French fries. Sample preparation was carried out as described by Becalski et al. [17], with a few modifications. A double SPE procedure was developed as follows. Briefly, about 1 g of the oil was dissolved in chloroform and acetone. Then, 100 μL of the sample was transferred onto the reverse-phase SPE cartridge (Waters Sep-Pak C18) (conditioned with methanol). For the simultaneous determination of GEs, a spiked extract was used as a mixture of internal standards, in a volume of 5 µL and at a concentration of 10 µg/mL. The sample was eluted with methanol under gravity at the rate of one drop per 2–3 s. The solvent was evaporated (at 40 °C in a nitrogen stream) to obtain a dry residue. Next, 55 µL of ethyl acetate was added to the residue in a 4-mL vial. The vial was capped and rotated to spread the solvent over the residue and was then left to stand for 5 min. Then, 0.95 mL of *n*-hexane was added to the vial, mixed well and left to stand for 5 min.

Normal-phase SPE cartridge (Waters Sep-Pak Silica) was conditioned with *n*-hexane/ethyl acetate 95:5 (v/v). Then, 1 mL of the sample extract was added to the cartridge and the eluate was collected following the previous procedures. The empty glass vial was washed using 2 mL of *n*-hexane/ethyl acetate and then the wash was applied to the cartridge. The process of washing the empty glass vial and transferring the solvent was repeated twice more (total 6 mL). The eluent was evaporated again to a dry residue. The residue was dissolved in 0.9 mL of diethyl ether and then the solvent was removed in nitrogen stream. An aliquot of 0.25 mL of methanol/isopropyl alcohol 1:1 (v/v) was added to the dry residue and the vial was mixed for 30 s. The solution was transferred to a micro-glass vial for LC–MS analysis.

The recovery % for the five standard spiked GEs were 99.5–103 % for 500 ng g^−1^, 98.5–102 % for 1000 ng g^−1^, 98.0–101.5 % for 2500 ng g^−1^ and 89–97.5 % for 12,500 ng g^−1^. The glycidyl ester content was obtained by using a Varian liquid chromatographic system (Paolo Alto, USA) consisting of a ternary pump (230 type Pro-Star series) and an autosampler (430 type Pro-Star series), as well as a degasser (MetaChem Technologies, USA). A single-quadruple mass spectrometer type 1200 L equipped with an atmospheric-pressure chemical ionization (APCI) interface was used. The analysis was conducted based on Granvogl and Schieberle [18], with a few modifications. The analysis was performedon a 150 × 2 mm i.d., Luna 3 μm PFP(2) 100 Å column equipped with a pre-column (Security Guard, 4 × 2 mm i.d.; Phenomenex, USA). The temperature of separation was 25 °C. The flow rate was set to 0.2 mL/min and the injection volume of the sample was 10 μL. Solvent A was 0.1 % formic acid in water, and solvent B was 0.1 % formic acid in acetonitrile. The mobile phase program was 80 % B, held for 15 min after injection, increasing the concentration of B to 100 % within 5 min, and holding again for 5 min. Then, the column was returned to 80 % B for 2 min and equilibrated for 8 min.

### Statistical Analysis

The data are presented as means ± standard deviations (SD) of duplicate technological experiments. Each analytical measurement was conducted in triplicate or five-fold (reflective index and color). Data handling and figure preparation was carried out using Excel 2007 (Microsoft, Seattle, WA, USA). The data were analyzed by two-way analysis of variance (ANOVA) using the Statistica 10.0 software. Duncan’s multiple range tests were used to determine the lowest statistically significant differences (LSD) between the means for *P* ≤ 0.05. The Pearson correlations between GEs and some of the examined parameters, as well as for the individual glycidyl esters and main fatty acids, were also investigated. Significance was determined at *P* ≤ 0.05. Regression analysis (*R* values) was designated using Excel 2007 (Microsoft).

## Results and Discussion

All oils were characterized by appropriate output parameters (Table 1) which indicated a good quality of oil [7, 8]. Thus, changes in these values during frying would indicate degradation of the oil’s quality.

**Table 1:** Characteristics of oils used as frying media

Kind of oil	Acid value (mg KOH g^−1^)	Peroxide value (meq O_2_ kg^−1^)	Anisidine value	Refractive index	*L*	*a*	*b*
RO	0.14 ± 0.01	0.46 ± 0.00	2.00 ± 0.85	1.46 ± 0.00	39.13 ± 0.01	−0.99 ± 0.10	10.40 ± 0.58
PO	0.10 ± 0.00	0.11 ± 0.00	3.00 ± 0.48	1.45 ± 0.00	38.56 ± 2.69	−1.73 ± 0.45	14.13 ± 0.24
POn	0.11 ± 0.01	0.07 ± 0.00	3.00 ± 0.29	1.45 ± 0.00	39.85 ± 0.34	−1.89 ± 0.30	15.13 ± 0.56
MIX	0.09 ± 0.01	0.16 ± 0.00	2.00 ± 0.25	1.45 ± 0.00	41.12 ± 0.78	−0.42 ± 0.02	8.63 ± 0.09

### Acid Value

**Fig. 1:**
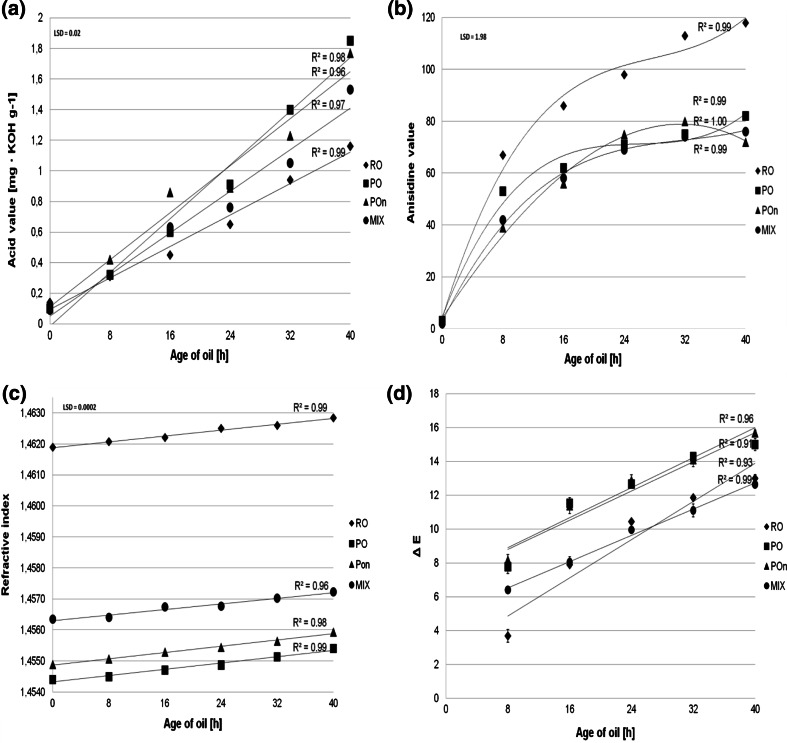
Changes of **a** acid value (mg KOH g^−1^), **b** anisidine value, **c** refractive index, **d** color (Δ*E*) in rapeseed oil (*RO*), palm oil (*PO*), palm olein (*POn*), and the blend (*MIX*) depending on the age of oil

Hydrolysis of frying oil triacylglycerols is one of the most common reactions that play a key role in frying oil degradation [19]. The linear increase of the free fatty acid content in frying media, regardless of the type of oil, was observed in all frying experiments (Fig. 1a). The most relevant changes in the free fatty acid content were observed in palm oil, and at the lowest estimate was in rapeseed oil. According to the Polish Directive from the Minister of Health of 25 September 2012 [20], the level of free fatty acids in frying medium higher than 2.5 mg KOH g^−1^ of fat cannot be acceptable. This level was not exceeded by any of the samples in our experiment. Other authors have also reported a linear increase in FFA content with frying time in different frying media [7].

### Anisidine Value

The secondary decomposition products are aldehydes formed during oxidative degradation and the non-volatile portion of the carbonyls remains in the frying oil. The use of the anisidine value is one of the most popular methods for monitoring oxidative stability. A rapid increase in AnV was observed for all frying oils during the first 3 days of frying as a function of time (Fig. 1b). Thereafter, increases in aldehyde contents were observed in most oils, excluding palm olein. After 32 h of frying, the AnV in palm olein decreased a little. Regardless of the general knowledge that the decomposition of hydroperoxides increases with increasing temperature and potentially the amount of carbonyls, AnV followed an opposite trend in this case. Carbonyl chemical reactivity, involvement in the formation of other compounds and thermal decomposition explains the decrease in AnV [21].

### Fatty Acid Composition

The fatty acid composition and chemical characteristics of the RO, PO, POn and also the blend (MIX) are shown in Table 2. The %SFA for the PO, POn and MIX was higher than that of the RO. The RO had the highest %PUFA, followed by the MIX, POn, and PO. The results of fatty acid composition indicate a progressive decrease in the contribution of linoleic acid, as the main polyunsaturated acid, throughout the frying period. Linoleic acid decreased by 9, 21, 23 and 28 % during 40 h of frying in RO, PO, POn and MIX, respectively. The ratio of linoleic acid to palmitic acid (C18:2/C16:0) has been suggested as a valid indicator of the level of polyunsaturated fatty acids (PUFA) determination. Our results showed the highest decrease in this ratio, from 4.55 to 3.38, in RO and the lowest from 0.20 to 0.15 in PO. In the study by Alireza et al. [22], the percent reductions of the ratio C18:2/C16:0 after 5 days of potato chips frying at 180 °C in palm olein/canola oil (1:1, w/w), palm olein/sesame/canola oil (1:1:1, w/w/w), sesame/canola oil (1:1, w/w), palm olein/sesame oil (1:1, w/w), palm olein and canola oil were 14.3, 15.4, 16.3, 20, 33.3 and 34, respectively. This confirms that the mixing of oil affects a noticeable change in the chemical reactions that occur during the frying process.

**Table 2:** Changes in contribution of fatty acid in rapeseed oil (*RO*), palm oil (*PO*), palm olein (*POn*), and the blend (*MIX*), depending on the type and age of oil

Age of frying oil (h)	Contribution^a^ (relative percentages)					
	C16:0	C18:0	C18:1	C18:2	C18:3	C18:2/C16:0
RO						
0 h	4.99 ± 0.06	1.85 ± 0.09	58.60 ± 0.38	22.71 ± 0.20	6.90 ± 0.06	4.55
24 h	5.36 ± 0.42	2.01 ± 0.13	59.86 ± 0.24	21.74 ± 0.14	5.54 ± 0.32	4.05
40 h	6.10 ± 0.14	2.08 ± 0.20	60.71 ± 0.73	20.63 ± 0.31	4.74 ± 0.61	3.38
PO						
0 h	46.62 ± 0.17	3.93 ± 0.20	36.76 ± 0.21	9.30 ± 0.39	0.26 ± 0.01	0.20
24 h	47.82 ± 0.02	4.12 ± 0.09	36.87 ± 0.06	7.94 ± 0.08	0.16 ± 0.01	0.17
40 h	48.11 ± 0.21	4.19 ± 0.12	36.94 ± 0.09	7.35 ± 0.20	0.16 ± 0.01	0.15
POn						
0 h	43.55 ± 0.62	3.96 ± 0.29	39.02 ± 0.28	10.50 ± 0.28	0.25 ± 0.03	0.24
24 h	44.47 ± 0.22	4.16 ± 0.21	39.13 ± 0.17	8.83 ± 0.17	0.23 ± 0.04	0.20
40 h	44.71 ± 0.36	4.41 ± 0.05	39.15 ± 0.23	8.05 ± 0.12	0.20 ± 0.00	0.18
MIX						
0 h	32.29 ± 0.42	3.58 ± 0.29	48.71 ± 0.48	11.26 ± 0.61	0.95 ± 0.13	0.35
24 h	33.75 ± 0.30	3.78 ± 0.33	48.80 ± 0.28	9.51 ± 0.43	0.59 ± 0.20	0.28
40 h	34.64 ± 0.32	3.81 ± 0.22	49.20 ± 0.00	8.16 ± 0.37	0.49 ± 0.12	0.24

^a^All values are averages ± standard deviation of triplicate analysis

### Refractive Index

One of the parameters used to determine the physical changes of the frying oils was the refractive index (RI) (Fig. 1c). RI is related to the fat autoxidation reaction, and its value increases after the formation of peroxides. In oils used in the experiment, with increasing frying time, RI increased in all frying media. However, the highest RI was observed in samples of RO.

### Color

Heating affected the color of oil across all frying variants. With increasing frying time, the *L* value decreased, while *a* and *b* values increased. The highest color changes were observed in RO after 40 h, while the most stable color was found in the blend (Fig. 1d). Similar results have been observed by other authors in rapeseed oil and their blends with palm olein, olive and corn oils during the frying process at 180 °C [7]. There was a report that darkening of the oil during deep frying is due to the polymer formation of unsaturated carbonyl compounds and non-polar compounds of foodstuff solubilized in the oil [23].

### Polar Compounds Content

The determination of polar compound content in frying oils provides the most reliable measure of the extent of oxidative degradation [24]. In this study, the contents of the polar fraction increased almost linearly with frying time, at the rate affected by the type of oil (Table 3). The total polar content during frying in RO was 17.45 ± 0.27 %, while in POn, it was 24.45 ± 0.89 % at the end of frying time. This was still below the 25 % (excluding one repeating of POn, which contained 0.08 % more than the limit) discard level set in many European countries, including Poland [20]. Farhoosh et al. [7] demonstrated a much higher rate of TPC formation. When rapeseed oil was used as a frying medium and blended with palm oil, the TPC level exceeded 25 % after 7.3 and 15.9 h of frying, respectively.

**Table 3:** Changes of polar fraction content and composition in rapeseed oil (*RO*), palm oil (*PO*), palm olein (*POn*), and the blend (*MIX*), depending on the type and age of oil

Type and age (h) of oil	Polar fraction content^a^ (g 100 g^−1^)	Polar fraction composition^a^ (contribution in relative percentages)					
		TGP	TGD	oxTAG	DAG	MAG	FFA
RO							
0 h	3.53^b^ ± 1.20i	ND	ND	51.15 ± 1.8a	31.36 ± 1.47g	ND	17.49 ± 0.64a
24 h	12.62 ± 0.45f	5.86 ± 0.23b	27.40 ± 0.28b	46.21 ± 0.14b	15.84 ± 0.14h	ND	4.69 ± 0.28b
40 h	17.45 ± 0.27d	9.02 ± 0.41a	34.95 ± 0.28a	42.55 ± 0.20c	9.41 ± 0.36i	ND	4.07 ± 0.10c
PO							
0 h	11.63 ± 0.13g	ND	0.97 ± 0.10j	12.49 ± 0.69i	85.32 ± 0.45a	0.54 ± 0.06ef	0.68 ± 0.10h
24 h	16.51 ± 0.16e	1.44 ± 0.14f	9.53 ± 0.12h	19.25 ± 0.13h	65.97 ± 0.29b	1.49 ± 0.11b	2.33 ± 0.23ef
40 h	21.31 ± 0.11b	2.48 ± 0.38e	14.01 ± 0336g	22.52 ± 0.39f	55.41 ± 0.25d	1.71 ± 0.13a	3.88 ± 0.17c
POn							
0 h	9.89 ± 0.14h	ND	0.80 ± 0.07j	11.83 ± 0.11i	85.10 ± 0.28a	0.70 ± 0.08d	1.58 ± 0.29g
24 h	18.44 ± 0.33c	2.25 ± 0.08e	15.36 ± 0.25f	19.00 ± 0.35h	60.69 ± 0.43c	0.79 ± 0.03d	1.91 ± 0.13fg
40 h	24.45 ± 0.89a	3.26 ± 0.40d	16.57 ± 0.32e	21.14 ± 0.20g	55.49 ± 0.28d	0.97 ± 0.04c	2.57 ± 0.34de
MIX							
0 h	9.30 ± 0.33h	1.59 ± 0.08f	2.22 ± 0.16i	12.25 ± 0.35i	84.98 ± 0.34a	0.23 ± 0.04g	1.57 ± 0.28g
24 h	16.47 ± 0.45e	5.05 ± 0.42c	17.71 ± 0.42d	24.22 ± 0.20e	50.84 ± 0.21e	0.49 ± 0.08f	1.70 ± 0.14fg
40 h	21.66 ± 0.35b	5.59 ± 0.40bc	19.41 ± 0.36c	26.96 ± 0.27d	44.39 ± 0.28f	0.65 ± 0.07de	3.01 ± 0.23d

*ND* not detected, *TGP* triacylglycerol polymers, *TGD* triacylglycerol dimers, *oxTAG* oxidized triacylglycerols, *DAG* diacylglycerols, *FFA* free fatty acids

^a^All values are averages of the triplicate analysis (*n* = 6)

^b^Values followed by different letters are statistically different at the 95 % confidence level

### Polar Compounds Composition

The composition of polar compounds formed during frying was analyzed using HPSEC (Table 3). Chemical changes occurring in fats and oils during frying involve the formation of a great variety of new compounds. Among them, TG dimers and oligomers are the major and more specific compounds [6]. In our study, the predominant fraction in fresh RO was oxidized triacylglycerols, with the presence of diacylglycerols and free fatty acids, while in palm oils, diacylglycerols significantly prevailed. During frying, new polar compounds were formed, including oligomers, which were at the highest level in RO after 40 h. We observed increasing levels of oxTAG, MAG and FFA and polymerization product concentration with decreasing levels of DAG during frying time in palm oils. Another trend with a decreasing level of oxTAG was reported in RO, which was in accordance with the findings of Aladedunye and Przybylski [21].

### Glycidyl Esters Content and Composition

Table 4 illustrates the GE levels in four frying mediums, as determined by the method reported in this study. The total amount of GEs detected in the fresh oils ranged from 0.80 to 25.34 mg kg^−1^ (for RO and POn, respectively). GE content similar to POn was determined in fresh samples of MIX (20.45 mg kg^−1^). During the frying procedures, the GE content decreased across all samples. Similar losses of GEs were found in palm oil and palm olein (44 and 38 % after 24 h, and 78 and 67 % after 40 h, respectively). In comparison, the losses of GE in RO at that time were 19 and 47 %, respectively. This indicates that the time at this range has only a minor impact on the GE content, and the main factor affecting this parameter is the kind of oil. Such results showed that GE degradation occurred almost twice as fast in the palm oils as in rapeseed oil. The results obtained show that, in the frying conditions, GEs that are present in the oil undergo destabilization. Hamlet et al. [1] noted that epoxides are very reactive compounds in which the epoxide ring can be opened by a variety of nucleophiles (acids, alcohols, water, amines, thiols, etc.). The epoxide ring can opened under both alkalic and acidic conditions although the rate of ring opening under the former conditions may be slower [1].

**Table 4:** Changes of composition of GE in rapeseed oil (*RO*), palm oil (*PO*), palm olein (*POn*), and the blend (*MIX*), depending on the type and age of oil

Type and age (h) of oil	GE content^a^ (mgkg^−1^)				
	C16:0-GE	C18:0-GE	C18:1-GE	C18:2-GE	Sum
RO					
0 h	ND	ND	0.57 ± 0.01hi	0.23 ± 0.02g	0.80 ± 0.01i
24 h	ND	ND	0.48 ± 0.02hi	0.17 ± 0.02gh	0.65 ± 0.01ij
40 h	ND	ND	0.42 ± 0.00i	0.00 ± 0.00i	0.42 ± 0.00j
PO					
0 h	2.19^b^ ± 0.09f	ND	2.92 ± 0.07f	0.70 ± 0.02e	5.81 ± 0.13f
24 h	0.93 ± 0.03g	ND	1.87 ± 0.10g	0.47 ± 0.08f	3.27 ± 0.20g
40 h	0.42 ± 0.02h	ND	0.67 ± 0.02h	0.18 ± 0.02gh	1.27 ± 0.06h
POn					
0 h	9.78 ± 0.03a	0.62 ± 0.02a	11.61 ± 0.00a	3.33 ± 0.08a	25.34 ± 0.07a
24 h	5.95 ± 0.09c	0.52 ± 0.04b	7.61 ± 0.25c	1.66 ± 0.15c	15.74 ± 0.02c
40 h	2.26 ± 0.05ef	0.00 ± 0.00c	2.97 ± 0.05f	0.79 ± 0.09e	6.02 ± 0.09f
MIX					
0 h	7.01 ± 0.02b	0.61 ± 0.02a	10.05 ± 0.10b	2.78 ± 0.22b	20.45 ± 0.29b
24 h	4.83 ± 0.19d	ND	6.29 ± 0.12d	1.48 ± 0.12d	12.61 ± 0.19d
40 h	2.42 ± 0.04e	ND	3.53 ± 0.13e	0.87 ± 0.14e	6.82 ± 0.03e

*ND* not detected (LOQ _C16:0-GE_ = 0.105 mg kg^−1^; LOD _C16:0-GE_ = 0.032 mg kg^−1^; LOQ _C18:0-GE_ = 0.125 mg kg^−1^; LOD _C18:0-GE_ = 0.045 mg kg^−1^; LOQ _C18:1-GE_ = 0.150 mg kg^−1^; LOD _C18:1-GE_ = 0.038 mg kg^−1^; LOQ _C18:2-GE_ = 0.095 mg kg^−1^; LOD _C18:2-GE_ = 0.028 mg kg^−1^)

^a^All values are averages of triplicate analysis

^b^Values followed by different letters are statistically different at 95 % confidence level

The GE content was also analyzed in fat extracted from fried French fries (Table 5). Semi products used in this study were frozen pre-fried French fries. Because of the exchange of the fat during frying between fried products and frying medium, the composition of oil used for pre-frying could influence the GE content of ready product. The higher GE content in fat extracted from French fries than in frying medium exhibited samples fried in used RO (after 40 h). This oil characterized by the lowest GE content in experiment and such situation is probably the results of fat exchange. The increased of GE content in fat from French fries could be influenced by quality of fat absorbed during first stage of frying. On the other hand GE content in fat extracted from fries fried in PO, POn and MIX was about one, five and three fold lower compared with fresh frying medium. That kinds of oils contained higher concentration of GE compared with RO. As a result of exchanging of oil, GEs could be absorbed with a frying medium into the product. As in the frying medium, GEs content in fat extracted from French fries decreased with frying time which could be the result of decomposition or conversion of GE in conditions of prolonged frying. After finishing of frying, the fat extracted from French fries fried in PO, POn and MIX characterized by about one and seven fold lower GEs content compared with used frying media.

**Table 5:** Changes of composition of GE in fat extracted from French fries fried in rapeseed oil (RO), palm oil (PO), palm olein (POn) and the blend (MIX), depending on the type and age of oil

Type and age (h) of oil	GE content^a^ (mgkg^−1^)				
	C16:0-GE	C18:0-GE	C18:1-GE	C18:2-GE	Sum
RO					
0 h	NDj	ND	ND	NDk	0.00 ± 0.00k
24 h	NDj	ND	ND	NDk	0.00 ± 0.00k
40 h	0.29^b^ ± 0.00i	ND	0.31 ± 0.00k	0.15 ± 0.00i	0.75 ± 0.00i
PO					
0 h	1.50 ± 0.00d	ND	2.07 ± 0.00c	0.31 ± 0.00c	3.88 ± 0.01c
24 h	0.76 ± 0.00f	ND	0.74 ± 0.00f	0.15 ± 0.00i	1.65 ± 0.00f
40 h	0.34 ± 0.00h	ND	0.30 ± 0.01h	NDk	0.64 ± 0.00j
POn					
0 h	3.60 ± 0.00a	ND	4.00 ± 0.00a	1.06 ± 0.00a	8.66 ± 0.00a
24 h	1.63 ± 0.00c	ND	1.43 ± 0.01d	0.28 ± 0.00d	3.35 ± 0.00d
40 h	0.44 ± 0.01g	ND	0.42 ± 0.01j	0.14 ± 0.00j	1.00 ± 0.00h
MIX					
0 h	3.48 ± 0.01b	ND	3.10 ± 0.00b	0.54 ± 0.00b	7.12 ± 0.00b
24 h	1.43 ± 0.01e	ND	1.31 ± 0.00e	0.25 ± 0.00e	2.99 ± 0.00e
40 h	0.34 ± 0.00h	ND	0.66 ± 0.01g	0.19 ± 0.00f	1.20 ± 0.00g

*ND* not detected (LOQ _C16:0-GE_ = 0.105 mg kg^−1^; LOD _C16:0-GE_ = 0.032 mg kg^−1^; LOQ _C18:0-GE_ = 0.125 mg kg^−1^; LOD _C18:0-GE_ = 0.045 mg kg^−1^; LOQ _C18:1-GE_ = 0.150 mg kg^−1^; LOD _C18:1-GE_ = 0.038 mg kg^−1^; LOQ _C18:2-GE_ = 0.095 mg kg^−1^; LOD _C18:2-GE_ = 0.028 mg kg^−1^)

^a^All values are averages of triplicate analysis

^b^Values followed by different letters are statistically different at 95 % confidence level

It was found that the glycidylester content in the frying oil at different levels of degradation was correlated with the main parameters describing thermo-oxidative changes in the oil during frying (Table 6). The GE content was significantly correlated with the acid and anisidine values and the refractive index. Significant correlations were also found between the main components of the polar fraction (diacylglycerols, oxidized triacylglycerol, dimers and free fatty acids). The strongest correlations were with the concentrations of diacylglycerols (*r* = 0.68) and oxidized triacylglycerols (*r* = −0.67).

**Table 6:** Significant correlation coefficients (*r*) between the sum of the glycidyl esters (GEs) and quality parameters of frying oil

Parameters of frying oil	Significant correlation coefficients (*r*)
Acid value	−0.43
Anisidine value	0.51
Refractive index	−0.49
Triacylglycerol dimers	−0.45
Oxidized triacylglycerols	−0.67
Diacylglycerols	0.68
Fatty acids	−0.44

C18:1-GE was found to be a prevailing GE of the highest concentration in all the studied samples, while C16:0-GE was the second major compound. This is in accordance with Weiβhaar and Perz [25], who suggested that, because of the fatty acid composition of palm oil, C16:0 and C18:1 esters are the most likely to be detected in the largest quantities. The glycidyl palmitate was correlated with palmitic fatty acid with a significant correlation coefficient, *r* = 0.42. In the analyzed oils, the samples also had C18:2-GE and C18:0-GE (excluding samples of rapeseed and palm oils), while not even a trace of C18:3-GE was found. In another study, samples were assayed for GE by 17 collaborating laboratories from seven countries. Commercial rapeseed oil was contaminated with C18:1-GE at a mean level of 0.45 ± 0.17 mg kg^−1^, which is similar to our results. Other GEs were not detected, whereas there were small amounts of C18:2-GE in our samples. Three palm oils were also examined. The C16:0-GE ranged from 2.34 to 2.36 mg kg^−1^ and C18:0-GE from 0.48 to 0.50 mg kg^−1^. The average C18:1-GE contamination showed values from 5.11 up to 5.37 mg kg^−1^, and for C18:2- GE it showed values from 1.33 to 1.39 mg kg^−1^. C18:3-GE was not detected in any analyzed samples [11]. In our study, we obtained similar results for C16:0-GE and C18:3-GE, but these were a half lower for C18:1-GE and C18:2-GE, and no trace of C18:0-GE was found, which could indicate a good quality of oil.

In frying conditions, with increasing frying time, a reduction of all the identified GEs was observed. In the samples of RO, the least stable was C18:2-GE, whose content after 24 h was characterized by a 25 % reduction, and at the end of the frying was degraded by 100 %. At the same time, the content of C18:1-GE decreased by 15 %, at about one-quarter of the initial value. In PO, more than half (57 %) of the C16:0-GE was degraded during the first 24 h, and, after a further 16 h of frying, was decomposed by more than 80 %. In comparison, esters of unsaturated fatty acids reduced their contents in the range from 33 to 36 % within 24 h and 74–77 % after 40 h of frying. In samples of POn and MIX, a similar rate of degradation was characterized for C18:2-GE: after 24 h, we observed its reduction at the level of 47 and 50 %, and, after 40 h, this was 69 and 76 % for MIX and POn, respectively. Degradation of C16:0-GE and C18:1-GE did not exceed 40 % after 24 h of frying in both oils, and, after 40 h, reductions of those GEs were10 % higher in POn than in the MIX. Shimizu et al. [26] conducted heating tests of pure diolein with various chloride and GE levels at 240 °C. They assumed that a part of GE seemed to be converted to 3-MCPD esters. In their opinion, raw materials for refining do not normally contain significant amounts of GE, and the practical impact appears to be small. We showed that the content of GE depends on the type of oil and palm oils characterized by a high concentration of those compounds. However, Shimizu et al. [26] notes that this pathway should be taken into consideration, because the form of 3-MCPD ester produced via GE is predicted to be a 3-MCPD monoester, which might show bioavailability different from the diester that mainly occurs in edible oils. It appears possible that the GEs can undergo reactions of decomposition or conversion after long deep frying, but there is no detailed data relating to those reactions. However, there are reports of research regarding the other compound present together with GE in refined oils. Ermacora and Hrncirik [27] prepared a study on the thermal degradation of 3-MCPD esters in model systems simulating deodorization of vegetable oils. They found that the formation pattern of the 2-MCPD diester followed the degradation of the original 3-MCPD diester. In a first stage, a quick formation of the 2-MCPD esters was observed after 2 h of thermal treatment, followed by a slow degradation over the remaining time period. Zhou et al. [28] examined the effects of temperature and water content on the formation of 3-MCPD esters in palm oil under conditions simulating deep fat frying but without frying of products. They concluded that 3-MCPD esters can be decomposed at high temperature when heating for a long time (longer than 2 h).

## Conclusions

Frying time and the type of frying medium affected the quality of oils used for the preparation of French fries. Hydrolysis and polymerization occurred most intensively in palm olein, while oxidation was most often reported in rapeseed oil. Frying conditions influenced the glycidyl esters (GE), which were newly-detected contaminants in refined oils. The content of GE was correlated with some chemical and physical parameters describing thermo-oxidative changes. The GE content decreased with increasing frying time. It seems that the initial level of GE is the main factor affecting the results. The highest degree of GE degradation was observed during frying in palm oil (78 %), while the lowest was obtained when frying was conducted in rapeseed oil (47 %). GE content in fat extracted from fried products was dependent on the frying medium in which they were prepared. The lowest concentration in fried French fries was characterized by rapeseed oil, and the highest in palm olein. In the works of other authors, GEs underwent reactions of decomposition or conversion after long thermal treatment, as well as 3-MPCD. This work presents a survey determining the effect of frying medium and time on oil degradation and GE content. The reduction of GEs that are found in many oil samples demonstrates that it would be interesting to know more about the degradation products of esters as well as their further transformation. In the long term, this could have a positive effect on reducing the exposure of humans to toxic compounds produced during the refining process.
